# Oral Lichen Planus: risk factors of malignant transformation and follow up. Ten years retrospective study

**DOI:** 10.4317/jced.57688

**Published:** 2021-07-01

**Authors:** Francesca Zotti, Riccardo Nocini, Giorgia Capocasale, Dario Bertossi, Andrea Fior, Martina Peretti, Erminia Manfrin, Massimo Albanese

**Affiliations:** 1 Researcher, DDS, PhD, Department of Surgical Sciences, Paediatrics and Gynaecology, University of Verona. Policlinico G. B. Rossi, Piazzale L. Scuro n.10, 37134, Verona, Italy; 2 Resident, MD, Department of Otorhinolaryngology - Head and Neck Surgery, University Hospital of Verona, Piazzale Aristide Stefani, n.1, 37126, Verona, Italy; 3 Resident, DDS, PhD, Department of Surgical Sciences, Paediatrics and Gynaecology, University of Verona. Policlinico G. B. Rossi, Piazzale L. Scuro n.10, 37134, Verona, Italy; 4 Associate professor, MD, MS, Department of Surgical Sciences, Paediatrics and Gynaecology, University of Verona. Policlinico G. B. Rossi, Piazzale L. Scuro n.10, 37134, Verona, Italy; 5 MD, MS, Unit of Dentistry and Maxillofacial Surgery, University Hospital of Verona, Italy; 6 Dental Hygienist, Private Practice, Verona, Italy; 7 Researcher, MD, MS, Department of Diagnostic and Public Health, University of Verona, Policlinico G. B. Rossi, Piazzale L. Scuro n.10, 37134, Verona, Italy; 8 Associate professor, MD, MS Department of Surgical Sciences, Paediatrics and Gynaecology, University of Verona. Policlinico G. B. Rossi, Piazzale L. Scuro n.10, 37134, Verona, Italy

## Abstract

**Background:**

Oral Lichen Planus (OLP) is an inflammatory chronic disease. Modified World Health Organization (WHO) diagnostic criteria (2003) suggest diagnosing OLP both clinically and histologically. Furthermore, it is known the potential of malignant transformation of OLP, especially those affecting mucosa. Aims of this retrospective study on 100 patients were i) to estimate the timing of transformation of OLP lesions in OSCC in a cohort of patients observed between 2008 and 2018; ii) to assess risk factors of OLP patients diagnosed with OSCC; iii) to analyse forms of OLP evolved in cancer.

**Material and Methods:**

A database of 100 patients diagnosed with OLP was evaluated and clinical, histological features of lesions, habits of patients and systemic diseases were analysed in a follow up ranged between 5 and 10 years.

**Results:**

Mean time of malignant transformation was 31,62± 18,26 months; however, 4 malignant transformations out of 8 occurred about after 4 years of observation. Furthermore, Odds ratios for risk factors showed an association between malignant transformation and location.

**Conclusions:**

More focused attention on follow-up scheduling and designing could be a valuable resource in early diagnosis and cancer prevention in OLP patients.

** Key words:**Oral Lichen Planus, Oral Cancer, Malignant transformation, risk factors.

## Introduction

Lichen Planus (LP) is a common idiopathic inflammatory disease of the skin and mucous membranes, it affects 1% to 2% of the general adult population, characterized by an autoimmune attack on the epidermis by skin-infiltrating T cells (1). This condition often occurs in different body areas involving skin, hair, nails, and mucosal (2,3).

Oral Lichen Planus (OLP) can affect several sites of the oral cavity (4-6) and several clinical manifestations are reported in the literature, according to Andreasen classification system (7): reticular (Wickham’s striae), papular, plaque-like, atrophic (erythematous), erosive-ulcerous and bullous-erosive; however, the classic lesions were bilateral, symmetric and with reticular pattern (1).

Diagnosis of OLP was controversial: Oral lichenoid lesions (OLL), Oral Lichenoid Dysplasia (OLD) and OLP show similar clinical and histologic features. However, OLL can occur as a result of contact sensitivity (e.g. for dental materials) or as a drug reaction and they tend to display a unilateral distribution, unlike the typical pattern of the OLP (bilateral and symmetric lesions) (8). Moreover, OLD is the term used to indicate oral dysplasia presenting histopathologic mimicry of OLP (9).

To overcome these problems, the diagnosis of OLP has to be made by taking into account both clinical and histological features, according to modified World Health Organization (WHO) diagnostic criteria (2003) (4).

As WHO considers OLP an oral potentially malignant disorder (OPMD), it is advisable to involve patients diagnosed with OLP in a periodic follow-up (10); this issue represents a further difficulty in managing the disease. Proven risk factors for the transformation of OLP lesions in OSCC are reported to be smoking, alcohol, erythematous lesions and their location in tongue margins (11-13).

However, the risk of OLP malignant transformation in Oral Squamous cellular Carcinoma (OSCC) remains debated: several prospective and retrospective studies argued about this issue reporting rates varying from 0–9 % (14-16).

Also, to date, the correct mechanism of OLP carcinogenesis remains unresolved, and then controversies still exist about the premalignant nature of OLP (16,17).

Aims of this retrospective study were i) to estimate the timing of transformation of OLP lesions in OSCC in a cohort of one hundred patients observed between 2008 and 2018; ii) to assess risk factors of OLP patients diagnosed with OSCC; iii) to analyze forms of OLP evolved in cancer.

## Material and Methods

This retrospective study was carried out evaluating histological records of one hundred confirmed cases of OLP, collected between January 2008 and December 2018 at the Department of Surgical Sciences, Paediatrics and Gynaecology, University of Verona. All procedures performed in studies involving human participants followed the ethical standards of the Institutional Research Committee (University of Verona, Italy) and with the 1964 Helsinki declaration.

All specimens derived from single or multiple-site oral incisional biopsies of different forms of OLP lesions.

According to our protocol, all the patients histologically and clinically diagnosed with OLP were involved in a follow-up to detect the potential transformation of lesions highlighted. During the follow-up visits, all anamnestic and clinical information were updated in medical records, where needed. Timing of follow-up visits was scheduled depending on the characteristics of lesions. Particular conditions, such as pain or ulceration, were evaluated in each case (1).

Nevertheless, at least two visits yearly for each patient were scheduled. During follow-up visits, clinical examination of the oral cavity and status of previous lesions were carried out. Moreover, adjunctive toluidine blue technique (18) was performed in OLP lesions of which clinical features appeared modified to plan eventual needed single or multiple-site incisional biopsies.

Only lesions firstly detected as OLP lesions were further histologically evaluated to assess malignant transformation.

Furthermore, at the first visit and during the observation period, photographs were taken with Nikon D200 camera (Nikon, Minato) equipped with Nikon AF-D DC 105mm f/2 lens (Nikon, Minato) and R1C1 dual flash (Nikon, Minato), both to have clinical features of the lesion and for recording the site in which biopsy sampling was performed.

In this study, we selected only cases in which OLP diagnosis was intended as both clinical and histological, according to modified WHO diagnostic criteria (2003) (4), to be careful to select only the cases of OLP and to exclude OLL and OLD. Data of patients diagnosed with OLP were extrapolated from a database. All other first histological and clinical diagnoses, included lichenoid dysplasia and lichenoid lesions, were not included to avoid selection bias.

After the inclusion/exclusion criteria assessment, the following information were researched in medical records and collected (19):

1. Patient demographics (age, gender).

2. habits (alcohol and smoking),

3. customary drug assumption and systemic diseases,

4. clinical features of lesions (20)

Our database of the photographs was also screened for pictures of selected patients.

In this study, an observation period of lesions between 5 and 10 years was considered, setting the end of our observation in 2018. The mean follow-up was 7 years and 6 months.

Timing of transformation of OLP lesions in OSCC was defined as the time between the first OLP diagnosis and the first histological diagnosis of OSCC and was evaluated and expressed in month ± SD. We considered only OSCC arisen in previously OLP lesion: we were able to evaluate this aspect through the photographs obtained at the first visit and during the observation period.

Risk factors evaluated were smoking, alcohol, erythematous lesions and location of them on tongue margins. The odds ratio was calculated to assess risk factors presence and evolution of OLP lesions in OSCC with an interval of confidence set at 95% (21). The hypothesis of an association between risk factors and the malignant transformation was tested using the Chi-square test, setting the significance level at 0,05.

A further statistical analysis was performed to assess the factors conditioning the malignant process (relative risk analysis). To analyse the relative risk for features of lesions, all erosive and ulcerative lesions were considered together.

Statistical analysis was performed using SPSS® Statistics 22 (IBM®, Armonk, North Castle, New York, USA).

## Results

A total of 100 patients with histological and clinical diagnoses of OLP were followed up between 2008 and 2018: 57 females and 43 males; they were aged between 27 and 86 years and the mean age was 62,5 years. Furthermore, 3% of patients were under 30 years, 6% between 30 and 39, 9% between 40 and 49, 22% between 50 to 59, 28% was observed in patients aged between 60 and 69 years and 23% in those aged between 70 and 79 years; a lower peak was 9% after the age of 80 years.

Smoking habit was also reported: 9 patients were smokers, 91 were non-smokers. No information about alcohol assumption was reported. Medical histories showed positivity for systemic diseases including hypertension, dyslipidemia, diabetes mellitus, thyroid gland disorder and three patients were positive for HCV.

In these cases, considering drug therapies, we had excluded suspected cases of OLL (8) through pictures.

Detailed clinical examination was reported for each patient to assess the location and clinical features of lesions. Patients showed oral lesions in multiple locations such as buccal mucosa, tongue, gingiva and labial mucosa. Moreover, in 7 patients, extra-oral manifestations were detected, 57,1% of those showed atrophic and erosive ulcers in the genital area; 42,9 % suffered from papular lesions of extremities and abdominal skin. In these cases, systemic administration of corticosteroids was initiated in conjunction with the Department of Dermatology.

The majority of lesions, 61, clinically appeared as plaque or papular/reticular, 39 were mixed atrophic/erosive with a plaque and papular/reticular aspect. Lesions colours were variable: 61% were white, and 39 % were mixed white/red.

A further biopsy, after the first histological confirmation, was performed in 21 patients; out of these, 14,3% (3 cases), were subject to an addiction third biopsy during the follow-up period.

Only one patient underwent more than three biopsies in 7 years.

All supplementary biopsies were performed due to procedural errors or clinical changes of lesions.

Those carried out within one month of the first biopsy were due to procedural errors, subsequent to inherent difficulties in performing this kind of surgery (inadequacy of tissue collected, undue tampering of the specimen). In particular, 21 adjunctive biopsies were performed: 4 of 21 adjunctive biopsies confirmed OSCC and 4 confirmed carcinomas in situ, therefore the malignant transformation rate was 8% of all diagnosed OLP patients involved in follow-up. All details of OSCC patients are shown in  Table 1.

**Table 1:**
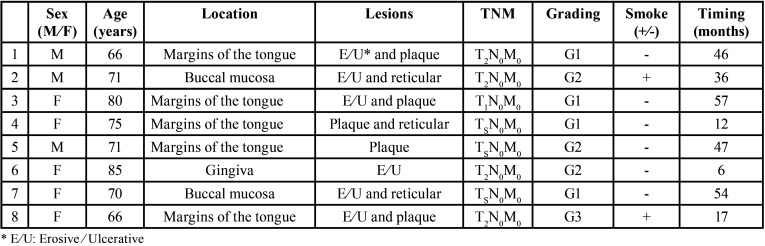
Overview of OSCC patients.

The mean period of malignant transformation resulted in 31,62± 18,26 months.

A particular case was represented by the only one patient developing cancer after 6 months and wearing an ill-suited mobile prosthetic denture (OSCC of gingiva associated with desquamative gingivitis and OLP histological confirmed diagnosis).

Moreover, one patient was periodically subjected to adjunctive multiple-site biopsies that showed dysplastic areas, according to the field cancerization phenomenon (22).

Risk factor analysis and odds ratios related were reported in  Table 2.

**Table 2:**
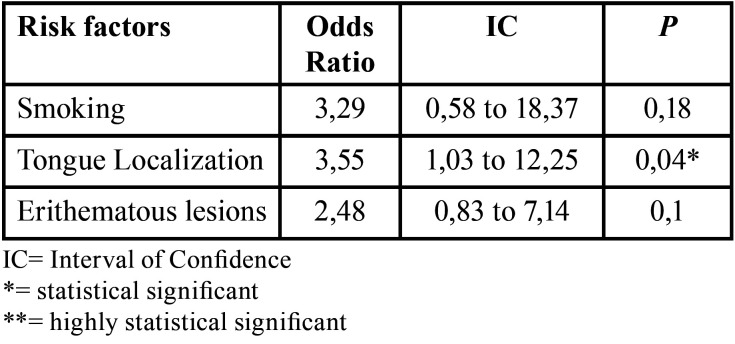
Chi-square analysis and odd ratios.

**Figure 1:**
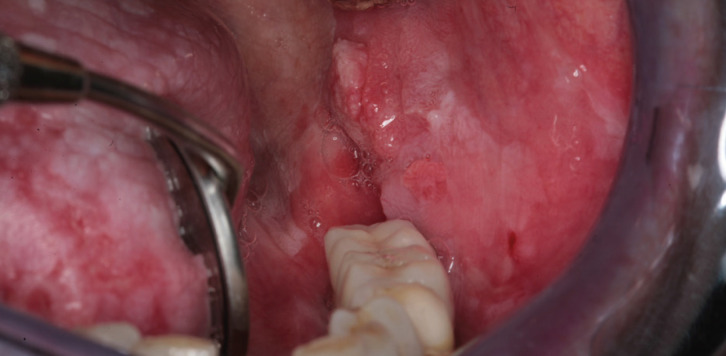
OSCC in a 70-years old female patient affecting the buccal mucosa associated with the typical clinical features of OLP. The lesions present as white striations (Wickham’s striae, reticular type) and erythema (atrophic) affecting the buccal mucosa and the tongue.

**Figure 2:**
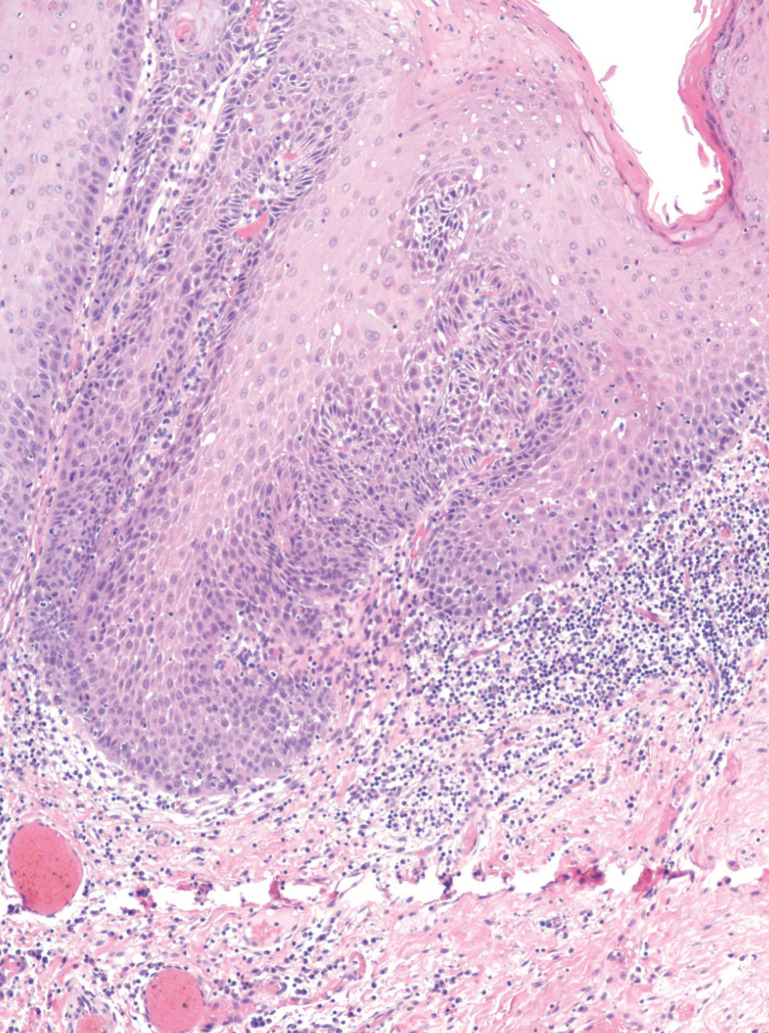
Band-like subepithelial lymphocytes in oral mucosa with epithelial hyperplasia that shows focal high grade epithelial dysplasia.

Erosive and ulcerative lesions and location on the margins of the tongue were found to be highly related to OSCC transformation (Figs. 1,2).

## Discussion

OLP is an inflammatory chronic disease involving both skin and mucosa, albeit skin lesions can become spontaneously asymptomatic, mucosal alterations do not heal and they cause symptoms and difficulties in their management (1). Transformation of OLP lesions in OSCC is surely the most significant complication of this disease; the literature reports several risk factors as responsible for malignant development. Mignogna M. *et al*. (23) described that OLP-related chronic inflammatory infiltrate is characterized by inflammatory factors often involved in cancer development. However, malignant transformation rate is a debated datum in the literature, especially because among studies there is no concordance in the definition of OLP diagnosis, specified by modified WHO diagnostic criteria (2003) (4). Furthermore, different PMDs can show similar clinical and histological features (OLL (24), Graft Versus Host Disease (25)) accounTable for inaccurate diagnoses and consequently for bias in clinical studies.

A systematic review and comprehensive meta-analysis of González-moles MÁ *et al*. (13) highlighted the recurrent bias of several studies, where dysplasia is not considered as a malignant transformation of OLP with a consequent alteration of rate’s values. Hence, the mean rate of malignant transformation is not sharp in literature due to diagnostic parameters used in OLP and because several studies consider malignant transformation the only developing of OSCC.

In our study, patients included fulfilled modified WHO diagnostic criteria (2003) (4) for OLP, however, as this is a retrospective study based on a search of histological records, the sample could have been underestimated. Moreover, based on this methodological design, all patients with an initial diagnosis of dysplasia were excluded, even if histological features were consistent with OLP, however, we considered dysplasia a malignant transformation. This choice led to a malignant transformation rate of 8% (26).

Data about the transformation rate of OLP lesions could be further influenced by the observation period of these lesions, namely by the follow-up of patients suffering from OLP. Because of this, the importance of this work is primarily found in the long-term follow-up evaluated: indeed in González-moles MÁ *et al*. (13), studies with shorter follow-up were reported (12 months), whereas we set the minimum observation period at five years with mean timing of malignant transformation of 31,62 ± 18,26 months. The choice to consider a so long follow-up, unlike several studies in the literature (27,28)(29), allowed to detect malignant transformations of OLP well after 3 years from the first diagnosis, this is a valuable datum to take into account for scheduling a follow-up in a prospective study.

However, we are well aware that malignant evolving can occur at any time, therefore a long-life follow-up is advisable.

In only one case we highlighted the development of OSCC in 6 months, however, this case deserves to be underlined because the patients reported chronic injury of prosthetic structure, not refitted despite the report, a risk factor still debated in the literature (30). The remaining patients were diagnosed with OSCC at ≥ 12 months.

Risk factors for malignant transformations were reported in the literature to be the location of OLP lesions (margins of the tongue), clinical features of lesions (red colour, heterogeneity) and incorrect habits (smoking, alcohol assumption). Here too conflicting data are present in the literature, Maher S *et al*. (15) reported malignant transformation in a handful of OLP patients, especially those smokers and alcohol consumers. On the other hand, in the review of González-moles MÁ *et al*. (13), the risk of malignant transformation was found to be 1.14%, increased where lesions are located on the margins of the tongue and with an erosive feature, associated with alcohol assumption and smoking. Based on this, we searched for these risk factors and we evaluated odd ratios reporting findings consistent with the literature. The odds ratio is a measure of association between exposure and an outcome and the confidence interval indicates the degree of uncertainty associated with the ratio. In this retrospective study, OR was used to calculate whether there was a correlation between the risk factors and OSCC developing in OLP patients. The strongest and statistically significant correlations were found with location on margins of the tongue (OR=3,55 and *P*=0,04), however, intervals of confidence deserve to be discussed. For tongue localization interval of confidence calculated was 1,03-12,25, suggesting that there was a 95% probability that the true odds ratio would be likely to lie in this range. Other risk factors all showed an odds ratio higher than 1 ( Table 2), indicating that risk factors were associated with higher odds of outcomes.

However, we would like to point attention to the interval of confidence and sample size in interpreting data and OR results and therefore highlight a limitation.

Another limitation of the present study in terms of risk factors is attributable to lack of information about alcohol consumption of patients involved because none of them reported drinking alcoholics, however, this datum seemed to be unlikely and therefore we could not estimate the risk of this factor.

We purposely avoided calculating in this work the potential association between age and sex and malignant transformation of OLP lesions because these findings were deeply assessed in literature (1); moreover, we decided to focus the attention on the extended observation period and the evaluation of risk factors.

An aspect deserving attention is the field cancerization (22), one patient of this study was subjected to periodical adjunctive multiple-site biopsies, which highlighted several dysplastic areas: based on this, we considered appropriate monitoring all lesions and keeping track of all biopsy sites by photographic records.

In literature emerged that several authors consider OLP as a PMD with a low malignant transformation rate and this aspect can lead to a reduction of focus during the OLP patients’ follow-up; nevertheless, the premalignant potential of OLP, the possible risk factors for such transformation, and the importance of follow up should be communicated to OLP patients.

Based on the evidence of malignant transformation after 4 years of observation in 4 cases of 8, it is intuitive the importance to extend follow-up of OLP patients, both in clinical routine and in designing prospective studies (26).

We were well aware that the data of this study were consistent with the literature and they seem to not increase the knowledge in this field. However, the log-lasted follow-up is the strongest point of this retrospective study because cancer transformation was found to appear also after 5 years of observation. This is, in our opinion, the main datum arising from this research and it should be taken into consideration when follow-up of OLP is set.

This consideration was confirmed by Fitzpatrick SG *et al*. (31): their review showed that the average time of malignant transformation was 51.4 months and therefore the authors recommended regular observation and follow-ups in patients with OLP or OLL.

In our perspective, multicenter studies should be carried out, particularly focused on diagnosis, risk factors (especially chronic injuries caused by prosthetic structures or restorations) and timing of follow-up. This might be of help in setting the malignant transformation rate and in standardizing the OLP patients follow-up taking into consideration clinical features, histological patterns, risk factors and systemic conditions. This issue is of great importance and it is widely studied in updated literature: for example, a meta-analysis by Idrees M *et al*. (32) showed that the rate of OLP malignant transformation is exaggerated in the studies included, mainly because of fails in the OLP diagnosis. This consideration was also recently reported by González-Moles MA *et al*. (33); ultimately, only prospective studies, carried out under strict methodological quality criteria could be of help in clarifying the correct malignant transformation rate.

Of course, another issue to take into account is the deep investigation of patients’ habits, especially alcohol and smoking. Knowing the correct anamnestic data could be diriment in scheduling proper follow-up visits, giving appropriate instructions to patients and therefore in early diagnosing malignant transformation.

An early diagnosis of OSCC is surely the only weapon capable to decrease mortality, morbidity, disfigurement, function loss, treatment duration, and hospital costs, therefore is an issue deserving all due care.
